# Biology and Feeding Behaviour of Ladybird, *Clitostethus arcuatus*, the Predator of the Ash Whitefly, *Siphoninus phillyreae*, in Fars Province, Iran

**DOI:** 10.1673/031.010.12001

**Published:** 2010-07-30

**Authors:** Z. Tavadjoh, H. Hamzehzarghani, H. Alemansoor, J. Khalghani, A. Vikram

**Affiliations:** ^1^ Department of Entomology, Science and Research Campus of Islamic Azad University, Tehran, Iran 14155-4933; ^2^ Department of Plant Protection, Shiraz University, Badjgah, Iran, 71444-65186; ^3^ Research Center of Natural Resources and Animal Husbandry of Fars province Shiraz, Iran 71555-617 and former faculty of Research Center of Natural Resources and Animal Husbandry of Fars province Shiraz, Iran 71555-617; ^4^ Evin Plant Disease and Pest Research Institute, Ministry of Jihad-e-Keshavarzi, Tehran, Iran 19395-1113; ^5^ Potato Development Centre, New Brunswick Department of Agriculture & Aquaculture, Wicklow, NB, Canada E7L 3S4

**Keywords:** biological control, population dynamics, Scymninae, Scymnini

## Abstract

*Clitostethus arcuatus* (Rossi) (Coleoptera: Coccinellidae) is considered as one of the most important natural biological control agents of the ash whitefly, *Siphoninus phillyreae* (Haliday) (Hemiptera: Aleyrodidae) in Iran. In the current survey, the development, survival, longevity, fecundity, feeding behaviour, and population dynamics of the predator under laboratory and field conditions were studied. The longevity of female insects was significantly longer than that of males. Total feeding of 4^th^ larval instars and females was significantly higher than males and other larval instars. The overall mortality rate from egg to adult under laboratory conditions was 22.7% while under field conditions it was 38.2%. Copulation lasted approximately 67 minutes while the average pre-mating and pre-oviposition times recorded were 3.8 and 1.8 days, respectively. The mean number of eggs laid by each female was 181. The adults could survive starvation for 4 days with a normal longevity of 62–73 days. The maximum population density of the predator was recorded in late August that coincided with the decline of the *S. phillyreae* population. *C. arcuatus* had four generations per year, and the adults were observed until mid December. Possible application of *C. arcuatus* for biological control of *S. phillyreae* in integrated pest management programs is discussed.

## Introduction

Whiteflies feed heavily on plant sap and produce sticky honeydew. Ash whitefly (*Siphoninus phillyreae*) (Halliday) (Hemiptera: Aleyrodidae) is a small white sap sucking insect that occurs in both temperate and Mediterranean climates. Nymphs and adults of this pest cause economic damage on the host plants mainly by feeding on plant sap from late May to mid December (Alemansoor and Fallahzadeh 2004). The highest population density of this pest occurs on ash trees, but more than seventeen other host plants (including apple, pears, apricot, acer, and plum) are reported under its host range in Fars Province of Iran (Alemansoor and Fallahzadeh 2004). It can also be found on many small trees and shrubs during outbreaks (Caon and de Barro 1999). Heavy infestations of *S. phillyreae* can cause stress to apple and pear trees, resulting in premature leaf drop, wilting, and smaller fruit size in Europe (Caon and de Barro 1999). Outbreaks of *S. phillyreae* frequently occur when their natural enemies have been disturbed or destroyed by pesticides or other factors (Flint 2002).

*S. phillyreae* has several natural enemies that can control its population to levels under the economic threshold. These natural enemies include predators such as *Clitostethus arcuatus* (Rossi) (Coleoptrea: Coccinellidae), *Menochilus* spp., and *Scymnus pallidivestis* and parasites such as *Coccophagus eleaphilus, Encarsia gautieri, Encarsia inaron, Encarsia partenopea, Encarsia siphonini, Encarsia pseudopartenopea; Eretmocerus siphonini,* and *Eretmocerus corni* (Nguyen and Hamon Avas 2000).

Among various parasitoids, predators and pathogens reported on *S. phillyreae*, the ladybird beetles have long been used as biological control agents (Onillon 1990; Obrycki and Kring 1998). In their recent studies, Alemansoor and Fallahzadeh (2004) reported *C. arcuatus* as a good candidate for controlling the pest in Fars. This ladybird beetle belongs to the subfamily Scymninae (the tribe Scymnini) and preys on species of Homoptera (Buntin and Tamaki 1980).

**Figure 1.**
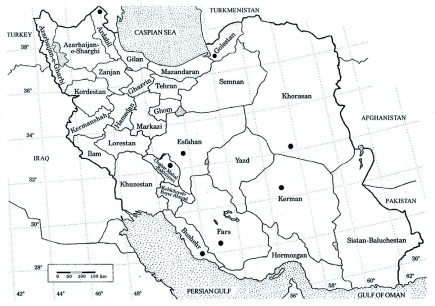
Distribution of *Clitostethu. arcuatus* in Iran. High quality figures are available online.

*C. arcuatus* is widely distributed in the Palaearctic region and was also reported from the former USSR (Agekyan 1977), Sardinia (Delrio et al. 1979; Ortus and Ibba 1985), Portugal (Magalhaes 1980), Africa (Fursch 1987), Italy (Gargani 1990), the USA (Bellows et al. 1990a; 1992b), Germany (Ziegler 1993), former Yugoslavia (Peric et al. 1997), Greece (Katsoyannos et al. 1998) and Iraq (Al-Alaf et al. 2001). In Iran, *C. arcuatus* was reported for the first time by Yazdani and Assadi (1989) in Fars Province and since then has been observed in other parts of the country (Lotfalizadeh 2001; Tavadjoh 2005) (Figure 1).

The predator prefers Aleyrodid species (Mills 1981) and has been used to control *S. phillyreae* in California. It preys on other species of Homoptera (Buntin and Tamaki 1980) including *Trialeurodes* *vaporariorum, Bemisia tabaci* (Gerling et al. 2001), *Dialeurodes citri* (Agekyan 1977; Basiri et al. 2004), *Aleurotrahelus jelinekii* (Mills 1981), and *Aleurodes proletella* (Bathon and Pietrzik 1986). In addition it also feeds on the eggs of *Tetranychus urticae* (Liotta et al. 2003) as well as aphids (Gerling et al. 2001).

A decline in the population of *S. phillyreae* was observed in all locations in Egypt after the release of the predator *C. arcuatus*. The population of *C. arcuatus* increased in all experiments following its release (AbdRabou 2006). The population of *C. arcuatus* spread over a wider area all the way from the northwest of Iran to coastal lines of Persian Gulf. Bathon and Pietrzik (1986) reported the colonization of the predator in the warmest regions of Central Europe. Although *C. arcuatus* is widely distributed in Iran, only a few studies have been conducted on the biology of this predator. The objective of this study was to investigate the biology and feeding behaviour of *C. arcuatus* as one of the potentially important biological control agents of *S. phillyreae*.

## Materials and Methods

### Rearing of *C. arcuatus*

**Figure 2.**
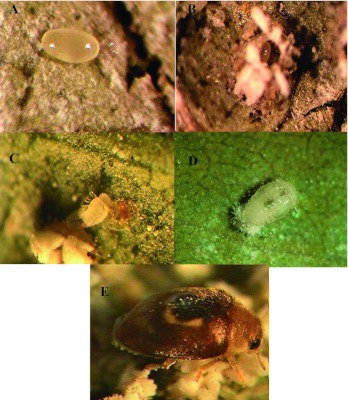
Developmental stages of the ladybird (*Clitostethus arcuatus*): Egg, A–B; Larva, C; Pupa, D; and Adult, E. High quality figures are available online.

Adults of the whitefly & *phillyreae* were collected from trees in nature, and a stand of 30 young ash trees in Shiraz were artificially infested with the pest. The infested trees were then maintained as a colony for further studies. The predator *C. arcuatus* (Figures 2A to E) was also collected from ash trees in April and were reared on young infested trees under field conditions. A culture of *C. arcuatus* fed on *S. phillyreae* on whitefly -infested ash leaves were reared in 51 mm polystyrene Petri dishes under laboratory conditions. The predators were additionally fed with syrup of honey made by adding one teaspoonful of honey in 100 ml water. Rearing containers were kept under laboratory conditions at 27 ± 1° C, 38 ± 2% RH, and a photoperiod of 16:8 L:D photoperiod.

### Biological studies conducted in the laboratory

Six pairs of newly emergent *C. arcuatus* were chosen, and each pair was placed separately in a Petri dish to evaluate daily oviposition and to measure their fecundity. Thirty five *C. arcuatus* eggs were transferred individually into separate Petri dishes to estimate the egg to adult mortality rate. The incubation period of the eggs and the lifetime of different larval stages, prepupae, pupae, and the adults were also recorded, with a difference for the latter in which only six pairs of adults were used. The mean duration of 36 pre-copulation times (the time between emergence of the adults and their first copulation) were recorded for twelve pairs of *C. arcuatus*. The time required for a copulated female to lay her eggs was also determined. One hundred pupae were collected from ash trees, and the number of males and females were recorded to determine the sex ratio.

### Biological studies conducted in the field

Six pairs of newly emergent predators were chosen and placed separately in a clip cage (6 cm in diameter) to evaluate the daily oviposition and fecundity. Thirty five eggs of *C. arcuatus* were transferred individually into thirty five clip cages mounted onto handpicked ash leaves with more or less evenly infestation to the ash whitefly to determine the egg to adult mortality rate. The incubation period of the eggs and lifetime of different larval stages, prepupae, and pupae were also recorded. The lifetime of the adults was recorded for six pairs using methods similar to those from the laboratory study. In order to determine the number of generations, twenty different stages of the *C. arcuatus* were kept under natural conditions in small separate clip cages and were then observed daily to determine their longevity and generation time. The number of *C. arcuatus* on five trees was counted for 15 min (between 9:45 and 10:00 am) every week from spring until late autumn in order to estimate the population density. The area around the host plants that were used for the field experiments was checked during winter to determine *C. arcuatus* hibernation sites.

### Feeding behaviour of *C. arcuatus*

Daily and the overall feeding of each active stage (larval and adult) of *C. arcuatus* were measured by placing 20 newly emergent samples of each stage in separate Petri dishes. Ten adults were randomly selected and placed in separate Petri dishes to investigate starvation tolerance and daily mortality of *C. arcuatus* adults. Daily observations were recorded from each adult without feeding them. To study the feeding behaviour, 15 adults were randomly selected from a population of *C. arcuatus* that had previously been starved for 24 hours and then exposed to 120 *S. phillyreae* eggs as a food supply. Subsequently, the feeding rate of each adult was recorded hourly for the first 6 h after adding the eggs and again at 12 h, 18 h, and 24 h.

**Table 1.**
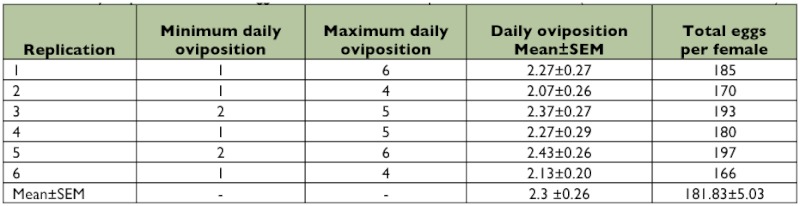
Daily oviposition and total eggs of *Clitostethus arcuatus* per female at 27 ± 1°C (*SEM*: Standard Error of Mean)

**Table 2.**
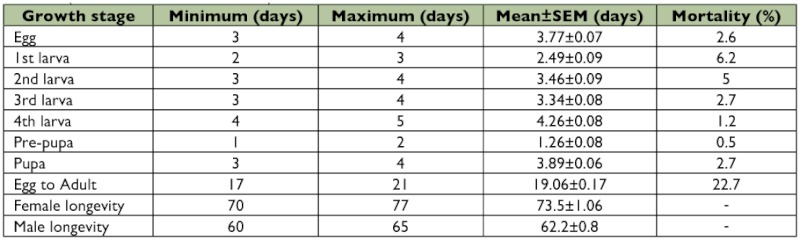
The longevity (day) and mortality (%) of different growth stages of *Clitostethus arcuatus* under laboratory conditions (*SEM*: Standard Error of Mean).

## Results

### Biology of *C. arcuatus* under laboratory conditions

The mean number of eggs laid per day per female was 2.3 ± 03 with a total of 181.8 ± 5.0 eggs per female during its lifetime (6 pairs) (Table 1). The mean duration of egg incubation, larval (1^st^, 2^nd^, 3^rd^, 4^th^) and pupal stages of *C. arcuatus* was 3.8 ± 0.1, (2.5 ± 0.1, 3.5 ± 0.1, 3.4 ± 0.1, 4.3 ± 0.1), and 3.9 ± 0.1 days, respectively (Table 2). The adults of *C. arcuatus* survived for 62– 73 days, and the predator lifetime under laboratory conditions was 19.1 ± 0.2 days. The highest mortality rate was recorded for the first larval instar (6.2%) and the mortality from egg to adult was 22.7% (Table 2). Pre-copulation time averaged 3.8 days, and the mean length of copulation time was 67.1 minutes. The results showed that the mated females laid their eggs after 1.8 ± 0.3 days. The females laid their eggs individually on the lower side of the leaf surface among *S. phillyreae* eggs. The *C.* *arcuatus* females preferred to lay their eggs close to the mass of *S. phillyreae* eggs and in the empty puparia of *S. phillyreaethrough* their exit holes left by emerging parasitoids from emerging parasitoid larvae (Figures 2A, 2B). The calculated sex ratio of *C. arcuatus* under laboratory conditions was found to be 1:1.38 (female:male).

### Biology of *C. arcuatus* under field condition

**Figure 3.**
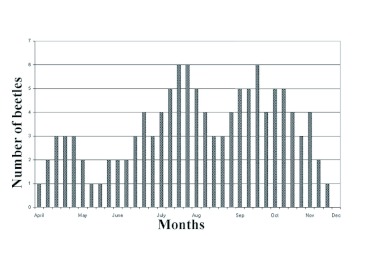
The population dynamics of the ladybird (*Clitostethus arcuatus*) on *Siphoninus phillyreae* in Shiraz in 2005. High quality figures are available online.

The mean number of eggs laid by *C. arcuatus* per day per female was 2.7 ± 0.5 with a total of 193.2 ± 5.6 eggs per female throughout its entire lifetime (Table 3). The mean durations of the egg incubation, larval and pupal stages of *C. arcuatus* are shown in Table 4. The adult *C. arcuatus* survived 63–77 days, and its lifetime under field conditions was 20.2 ± 0.1 days. The mortality of the egg-adult period for *C. arcuatus* was 38.2%. *C. arcuatus* was commonly active for eight months (from April to December) in the Shiraz region, having four generations per year. The population density of the predator steadily increased from mid July until it reached a first main peak in mid August and declined gradually followed by another increase leading to a second main peak in mid October (Figure 3). *C. arcuatus* usually prepares for hibernation in mid December when the ash leaves begin to shed, and their hibernating areas include host plant debris, clods of earth, and the cracks of tree barks. It played a significant role in the reduction of the *S. phillyreae* population and showed a capacity to suppress the *S. phillyreae* population as its population increased during summer months.

**Table 3.**
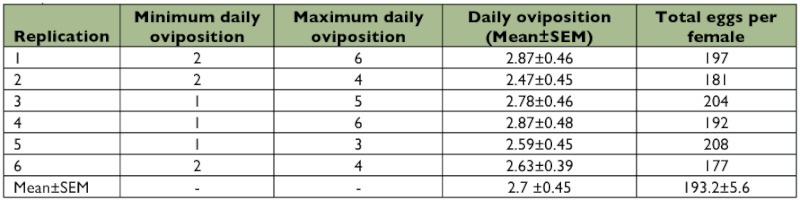
Daily oviposition and total eggs per female of *Clitostethus arcuatus* under field conditions (*SEM*: Standard Error of Mean)

### Feeding behaviour of *C. arcuatus*

Feeding by *C. arcuatus* started 15 min after eclosion of the first larval instar, and the adults can consume an egg in 15–20 s. Newly hatched 1^st^ instar *C. arcuatus* larvae were relatively immobile and fed on *S. phillyreae* eggs and nymphs over a limited leaf surface area. The 2^nd^, 3^rd^ and 4^th^ larval instars of *C. arcuatus* moved rapidly on the leaf surface and fed on all nymphal stages of the host while showing a preference for eggs. Total feeding by larva of *C. arcuatus* amounted to 259 ± 3 *S. phillyreae* eggs The total number of eggs consumed by males and females of *C. arcuatus* was 2024 ± 39 and 4023 ± 74, respectively (Table 5).

**Figure 4.**
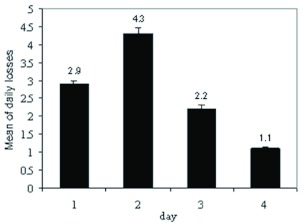
Daily losses in adults of *Clitostethus arcuatus* during the starvation period. Values are the means of four replicates; error bars represent standard error of the mean. High quality figures are available online.

**Figure 5.**
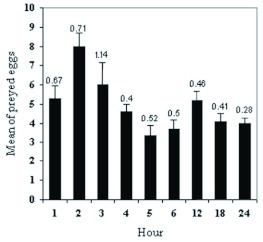
Feeding behaviour of *Clitostethus arcuatus* in a 24-h period. Values are the means of four replicates; error bars represent standard error of the mean. High quality figures are available online.

The adults of *C. arcuatus* survived starvation for a maximum period of 4 days. The greatest loss of adult *C. arcuatus* due to starvation occurred in the second day (Figure 4). To assess feeding rate of the predator, 15 adult *C. arcuatus* were randomly selected from a population that had previously been starved for 24 hours and exposed to 120 *S. phillyreae* eggs as a food supply. The consumption of eggs was recorded hourly for the first 6 h after feeding and again at 12 h, 18 h, and 24 h after feeding. The results showed that highest and lowest feeding activity occurred in the 2^nd^ and 5^th^ hours, respectively. There was a sharp increase in feeding in the 2^nd^ hour after feeding followed by a steady decline until the 5^th^ hour where the feeding rate dropped to its minimum and began to increase slowly to reach to a second smaller peak at 12 hours after feeding, which was followed by a less steep increase. The differences observed between feeding rates of starved predators at different hours after feeding were highly significant (Figure 5).

**Table 4.**
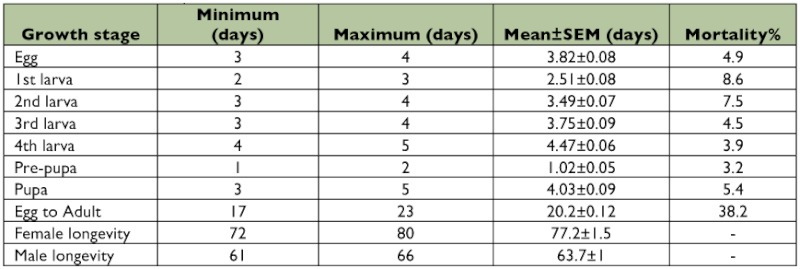
The longevity (day) and mortality (%) of different growth stages of *Clitostethus arcuatus* under field conditions (*SEM*: Standard Error of Mean).

**Table 5.**
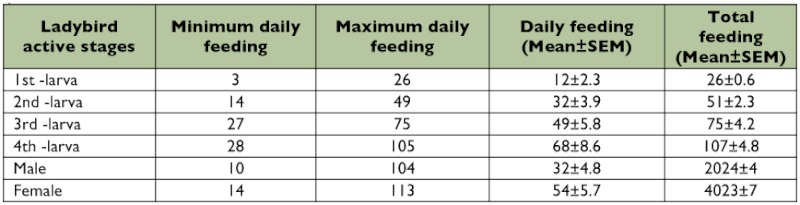
Daily and total feeding of eggs in the larval instars and adults of *Clitostethus arcuatus* (*SEM*: Standard Error of Mean).

## Discussion

Biological studies on *C. arcuatus* showed that the mean duration of egg development, larval and pupal stages were 3.8, 11.4 and 3.9 days, respectively. Egg incubation time was relatively shorter than the value reported by Agekyan (1977), which might be attributed to the fact that the host *Dialeurodes citri*, was different. From our studies *C. arcuatus* adults survived for 62– 73 days on *S. phillyreae* eggs, which is considerably lower than the survival of *C. arcuatus* on *Aleurodes proletella*, which was reported to be approximately 150 days (Liotta 1981). The longevity of females (73 days) is was higher than males (62 days), which is consistent with the results of other studies (Bellows et al. 1992).

The first larval instar had the highest mortality rate, and the mortality of egg to adult was 22.7%, which is consistent with results from Bellows et al. (1992), who reported egg to adult survival of 78% (Table 6). A higher mortality rate for the first larval instar is conceivable because this instar is the most vulnerable stage for *C. arcuatus*, followed by the 2^nd^, 3^rd^ and 4^th^ instars. Mortality of *C. arcuatus* stabilizes after the 2^nd^ instar. Immature instars of higher stages survive adverse environmental conditions better and accordingly have a lower mortality rate.

Egg mortality, while high, was still lower than the mortality of the first instar. “The eggs were laid mainly in the empty puparia of *S. phillyreae* through the exit holes left by emerging parasitoids which might provide more protection for the eggs. Our results also showed that the development from egg to adult required an average of 15.6 days and that the average survival ability of the females was 82 days. The results of the experiments were produced under laboratory conditions (27 ± 1° C, 38 ± 2% RH and 16:8 L:D) which was close to the optimum temperature (28.2° C) for the development, survival, and fertility of *C. arcuatus* reported by Bellows et al. (1992).

Our studies reported a lower sex ratio (1:1.38) for *C. arcuatus* as compared to the sex ratio (1:1) reported by Bellows et al. (1992).. In addition, the mean number of eggs laid per day per female in present experiments was 2.3 ± 0.3, with a total of 182 ± 5 eggs per female throughout its entire lifetime, which is lower than the 202 eggs per day per female reported by Bellows et al. (1992) (Table 6).

Genetic heterogeneity of the local populations of *C. arcuatus* added to the inherent demographic stochasticity of *C. arcuatus* individuals and possibly the use of *S. phillyreae* as prey may account for minor inconsistencies between our results and other findings. *C. arcuatus* is normally active for eight months, from April to December, in the Shiraz region where it has four generations per year. Although the number of generations per year was quite close to that reported from Italy (Liotta 1981), the study conducted in Germany reported three generations per year (Bathon and Pietrzik 1986).

**Table 6.**
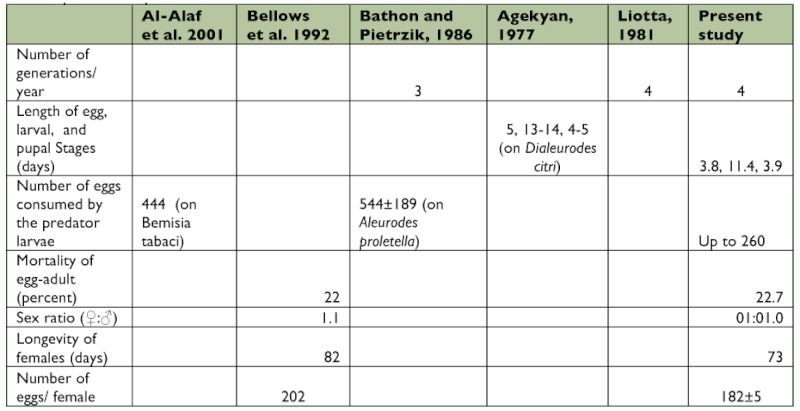
Comparison of records of biological and feeding behaviour biometrics of the predator *Clitostethus arcuatus* between present study and results of other studies

The larvae of *C. arcuatus* feed mainly on *S. phillyreae* eggs and nymphs, and possibly adults (Bathon and Pietrzik 1986). They can also feed on some species of Tetranichidae (Yazdani and Assadi 1989). *C. arcuatus* larvae puncture the eggs or nymphs of whiteflies using their mouthparts and suck their contents. This type of feeding behaviour is a characteristic feature of *Scymnus* species (Buntin and Tamaki 1980; Brown et al. 1995).

Our results show that under laboratory conditions, each larva of *C. arcuatus* is capable of consuming of up to 260 *S. phillyreae* eggs during its life time. It has *A. proletella* in central Europe where each larva consumes about 544 ± 189 eggs during its lifetime (Bathon and Pietrzik 1986). *C. arcuatus* larvae are reported to consume an average of 444 whitefly eggs on *B. tabaci* (Al-Alaf et al. 2001). Each male and female consumes about 27.4 and 60.7 whitefly eggs per day, respectively (Bathon and Pietrzik 1986). Agekyan (1977) observed that *C. arcuatus* has a preference for eggs and 1^st^ instar nymphs of *S. phillyreae*. Lower consumption rates of the eggs of *B. tabaci* (approximately 444) *C. arcuatus* larvae (Al-Alaf et al. 2001) may be accounted for by host differences and differential egg consumptions by males and females, which are 27.4 and 60.7 eggs of *A. proletella* per day, respectively (Bathon and Pietrzik 1986). Further research on this will certainly be required to clarify the host effect on the biology and feeding behaviour of *C. arcuatus* (Table 6).

In this study, the larval and adult stages of *C. arcuatus* showed cannibalistic behaviour. The larvae fed on conspecific eggs, younger instars, and even pupae, while the adults fed mainly on the eggs. Similar observations on cannibalistic behaviour of different stages of *C. arcuatus* have been reported from Italy (Liotta 1981).

Natural enemies have a central role in successful reduction of the economic damage levels of pests and attempts should focus on an integrated approach that can employ a diverse range of tactics to help produce an environment favourable to them. A reasonable approach toward the control of ash whitefly, *S. phillyreae* might be to look for natural enemies that will become established and lower the pest population in the wild. Based on these findings, the predator *C. arcuatus* has a relatively short lifetime and rapid reproduction rate (four generations per year) and can be considered a potentially important biocontrol agent in Iran. Integrated & *phillyreae* management requires consistent and reliable data on the biology of this key predator. A comparison of these findings on *C. arcuatus* show some differences with the results of the few other studies conducted on its biology.

The results obtained from the data collected in field studies are often preferred and more reliable since they account for the effect of numerous unknown influencing factors that could be controlled otherwise in a laboratory study. However, for the same reason, lower accuracy of the results from field observations is common. Alternatively laboratory studies produce results that are consistent and reproducible, but they do not reproduce a comprehensive image of the inherent natural randomness of the reality. Neither the lab nor the field studies are perfect alone, and hence this study benefited from a combination of both laboratory trials and field observations.

Further research both in the laboratory under controlled conditions and in the field is required to study the possibility of mass rearing and application of *C. arcuatus* as a candidate for biological control of *S. phillyreae* in integrated pest management programs, especially under greenhouse conditions.
